# Designing, conducting, and reporting reproducible animal experiments

**DOI:** 10.1530/JOE-22-0330

**Published:** 2023-06-19

**Authors:** Emma Wilson, Fiona J Ramage, Kimberley E Wever, Emily S Sena, Malcolm R Macleod, Gillian L Currie

**Affiliations:** 1 Centre for Clinical Brain Sciences, The University of Edinburgh, Edinburgh, UK; 2 Simons Initiative for the Developing Brain, The University of Edinburgh, Edinburgh, UK; 3 Department of Systems Medicine, School of Medicine, University of Dundee, Dundee, UK; 4 Department of Anesthesiology, Pain and Palliative Care, Radboud University Medical Center, Nijmegen, The Netherlands

**Keywords:** reproducibility, open research, animal research, experimental design

## Abstract

In biomedicine and many other fields, there are growing concerns around the reproducibility of research findings, with many researchers being unable to replicate their own or others’ results. This raises important questions as to the validity and usefulness of much published research. In this review, we aim to engage researchers in the issue of research reproducibility and equip them with the necessary tools to increase the reproducibility of their research. We first highlight the causes and potential impact of non-reproducible research and emphasise the benefits of working reproducibly for the researcher and broader research community. We address specific targets for improvement and steps that individual researchers can take to increase the reproducibility of their work. We next provide recommendations for improving the design and conduct of experiments, focusing on *in vivo* animal experiments. We describe common sources of poor internal validity of experiments and offer practical guidance for limiting these potential sources of bias at different experimental stages, as well as discussing other important considerations during experimental design. We provide a list of key resources available to researchers to improve experimental design, conduct, and reporting. We then discuss the importance of open research practices such as study preregistration and the use of preprints and describe recommendations around data management and sharing. Our review emphasises the importance of reproducible work and aims to empower every individual researcher to contribute to the reproducibility of research in their field.

## Introduction

Animal research is critical to further our understanding of the biological phenomena which underpin human diseases and their treatment. To benefit human health, research findings must be robust and reliable. Many attempts to reproduce the findings of animal experiments have failed, prompting some researchers to argue that science is in the midst of a reproducibility crisis (Begley & Ioannidis 2015).

Reproducibility can be defined as a complex concept encompassing a continuum of research processes: ‘reproduction’, ‘replication’, and ‘re-use’. Each of these relies on transparent reporting and the availability of data (European Commission, Directorate General for Research and Innovation 2020). In this review, we will use the broad definition of reproducibility as the ability to obtain results consistent with an original specific finding.

Various factors may contribute to irreproducibility (Macleod & The University of Edinburgh Research Strategy Group 2022), including weaknesses in experimental design, conduct, analysis, and reporting. Such weaknesses raise concerns about the credibility of the ‘foundations’ of knowledge on which further research and exploitation of research findings are based. Strong efforts have been made to identify and systematically evaluate the causes of and impact of irreproducibility in animal research (summarised in (Russell *et al.* 2022)). These efforts have led to the development of many tools and guidelines, including the PREPARE guidelines for planning animal experiments (Smith *et al.* 2018), the EQIPD framework and quality system for the conduct of experiments (Bespalov *et al.* 2021, Vollert *et al.* 2022) and the ARRIVE guidelines for reporting of research using animals (Percie du Sert *et al.* 2020), which together aim to improve transparency, reproducibility, and experimental animal welfare. However, for these tools to have their desired effect, researchers must first be aware of them, and secondly, be able to incorporate them into their day-to-day practice.

This review aims to raise awareness of the impact of irreproducibility in animal research, advocate simple methods to improve reproducibility, and signpost researchers towards existing tools and guidelines available to help them. We stress that while improving reproducibility can appear to be a substantial challenge, researchers can begin to make a difference by taking simple steps to work more reproducibly.

## Reproducibility and research

### The impact of irreproducible research

The impact of non-reproducible findings is a pressing topic within research. Estimates of the proportion of irreproducible findings within biomedical research range from 70 and 90% (Begley & Ellis, 2012, Begley & Ioannidis 2015, Freedman *et al.* 2015, Baker 2016). Consequently, it is estimated that half of the preclinical research funding (with a conservative estimate of $28B/year in the United States alone) may be spent on research findings which are not reproducible (Freedman *et al.* 2015). Beyond financial and resource concerns, the persistence of non-reproducible findings in the literature may mislead the planning of future research (Begley & Ellis 2012), generating further downstream research waste.

### The benefits of working reproducibly

All researchers should strive to produce work of the highest quality. Alongside this obligation, there are many benefits to taking steps to improve the reproducibility of their work. Improved research efficiency and animal welfare serve to benefit the wider scientific community. However, working more reproducibly also benefits individuals by allowing a greater likelihood of picking up errors prior to publication, more efficient manuscript writing, fewer problems during peer review, and reputational benefits (Markowetz 2015).

### Irreproducible research thrives in a negative research culture

To better understand irreproducibility and its impact on research, we must first consider the factors that may contribute towards irreproducibility, including the effect of research culture, and approaches to experimental design, conduct, analysis, and reporting.

The prevalence of studies of poorer methodological quality may be partly due to a research culture which manifests distorted research incentives, with strong emphasis placed on publishing ‘exciting’, novel results, with outcomes such as funding and career progression hinging on a constant output of impactful research (Munafò *et al.* 2017, Macleod & The University of Edinburgh Research Strategy Group 2022). In a survey conducted by *Nature*, over 60% of researchers claimed that pressure to publish and selective reporting of results contributed to irreproducibility in their field (Baker 2016). A more recent survey shows that although extreme research misconduct such as fabrication is uncommon, questionable research practices that may be detrimental to the research process are not (Xie *et al.* 2021).

While there is a vast array of support and educational materials available to help researchers improve the reproducibility of their experiments (see Table 2), education on reproducibility is not always incentivised or a formal part of mandatory training (Macleod & The University of Edinburgh Research Strategy Group 2022). Additionally, a lack of awareness of existing resources may contribute to their limited use.

### Targets for improvement

A more optimistic viewpoint considers ‘failed’ reproducibility as an opportunity to engage researchers and encourage continuous improvement to research standards, rather than as a crisis that can and must be resolved (Munafò *et al.* 2022). The ‘reproducibility opportunity’ approach prompts the adoption of more reproducible methodologies and workflows, promoting best standards and practices, leading towards improved scientific rigour and reproducibility.

The reproducibility of animal research could be improved through systemic top-down approaches from institutions, funding bodies, and publishers, aimed at significantly and meaningfully improving the research environment (Macleod & The University of Edinburgh Research Strategy Group 2022). Several such approaches have already been implemented or suggested, including DORA (the Declaration of Research Assessment), which aims to improve the way research outputs are evaluated (Cagan 2013), and results-blind peer review, which aims to mitigate publication bias (i.e. the phenomenon that ‘positive’ results are more likely to be published than ‘negative’ or ‘neutral’ findings (Locascio 2017)).

However, we stress that there is also much that researchers can do at an individual level via a bottom-up approach, by taking small steps to improve the reproducibility of their own work. The remainder of this review will focus on such efforts, with the intention of empowering individual researchers by providing important considerations relevant to their research and signposting towards resources and tools which have been developed specifically to help animal researchers to improve the quality of their work.

## Designing reproducible animal experiments

### Factors which influence study validity

Several factors, including flaws in experimental design, research materials, methods and technical considerations, data management and analysis, and transparent reporting, may be contributing to irreproducibility (Landis *et al.* 2012, Freedman *et al.* 2015). The Landis criteria are four essential recommendations that can help researchers improve the reproducibility of their work (Landis *et al.* 2012). We build on these recommendations to give further advice on experimental design, including complexity, planning, controls, and biological variables.

It is worth noting that to implement these solutions, discussions should take place with those involved in the planning of experiments and care of research animals including animal house personnel and local veterinarians. This review should help facilitate these discussions.

### Internal validity and risks of bias

When an experiment has high internal validity, it is likely that any effect observed in an experimental group is due to a true effect observed in the experimental population rather than to methodological error or bias, influenced by researcher behaviours or preconceptions. Factors which could threaten internal validity include bias in the allocation of animals to experimental groups (selection bias), systematic differences in how animals in different experimental groups are handled or cared for apart from the intervention being tested (performance bias), systematic differences in how outcomes are assessed in different groups (detection bias), and the unequal handling of animal drop-outs between treatment groups (attrition bias) (van der Worp *et al.* 2010). A summary of how certain types of bias can be introduced in animal experiments, alongside recommended steps to reduce these biases, is given in Table 1.

**Table 1:** Summary of some of the actions which may introduce bias and reduce the internal validity of an experiment, the types of biases they introduce, and solutions to avoid these biases.

Threat to internal validity	Bias introduced	Solution	Practical advice
Biased allocation of animals to experimental groups	Selection bias	Randomisation	✓ Do randomly allocate animals to experimental groups using a random number generator (several online tools are available).
			✓ If there are key baseline characteristics which need to be evenly distributed across the groups, conduct randomisation in blocks according to those key baseline characteristics.
			✘ Do not select animals haphazardly or serially, for example, by picking them one by one from a cage and assign to groups or allocating all animals of the same sex or from the same litter to a group.
			✘ Do not move animals between groups even if they did not receive the assigned treatment. The random sequence determines the group allocation. Animals should still be considered a member of their allocated group, and their outcome data (if available) analysed as such. A secondary ‘on-treatment’ analysis is allowable but carries less weight.
		Blinding	✓ Do randomly allocate animals to experimental groups without knowing which group will be receiving which treatment (e.g. label groups 1, 2, and 3).
			✓ Do ask have group allocation conducted by a third party (e.g. a colleague not otherwise involved in the experiment); they should store unblinded allocation securely, in a place not available to other staff involved in the experiment.
			✘ Do not use informative group labels (e.g. do not label the groups ‘exp’ and ‘ctrl’). The allocated group labels should be non-informative (i.e. coded alphanumeric identifiers).
Systematic differences in how animals in different experimental groups are handled or cared for apart from the intervention being tested	Performance bias	Randomisation (performance)	✓ Do perform any interaction with the animals in a randomised order across groups. If animals were properly randomised at allocation and are only identifiable by their number (see later), the order of the animal number can be used.
			✘ Do not perform interactions with the animals in sequence according to their group allocation, for example, sacrificing all animals in group 1 in the morning and group 2 in the afternoon.
		Blinding (performance)	✓ For optimum blinding, label animals only with an alphanumeric identifier (e.g. rats 1A–26X) and avoid being aware which animals belong to the same group. Being aware which animals belong to the same group can cause blinding to fail if, for example, patterns in behaviour become apparent.
			✓ Less optimally, use partial blinding to mask group labels, for example, assign animals to groups ‘A’ and ‘B’ rather than ‘treatment’ and ‘control’.
			✓ Do, if handling or treating the animals requires a member of staff to be unblinded, exclude that person from participating in any other phase of the experiment.
			✓ Do make any decisions on animal welfare concerns blinded to group allocation.
			✘ Do not assign animals to openly labelled groups, for example, ‘treatment’ and ‘control’.
Systematic distortion of the results of a study that occurs when the outcome assessor has knowledge of group allocation	Detection bias	Blinding (outcome assessment)	✓ Do use blinding at the time of outcome assessment, with the help of research assistants or technicians if needed:
			► (Gold standard): label animals only with their alphanumeric identifier and avoid being aware of which animals belong to the same group.
			► (Second best, partial blinding): mask group labels.
			✘ Do not assume that any outcomes can be assessed – whether behavioural or otherwise – in an unbiased unblinded manner.
			✘ Do not remove blinding until analysis is completed. For studies with alphanumeric labels only, first unblind the grouping (i.e. to group A, group B), then perform the analysis, then unblind the identity of groups A and B).
Unequal handling of animal drop-outs between treatment groups	Attrition bias	Pre-define and report exclusions and their criteria	✓ Do pre-define the criteria for any exclusions.
			✓ Do make sure that exclusion criteria are not directly related to the condition or treatment being studied.
			✓ Do ensure that the individual responsible for the decision to exclude an animal is external to the experiment and has no knowledge of group allocation (e.g. the house veterinarian).
			✓ Do clearly report the total number of exclusions and reasons for exclusion for each experimental group.
			✘ Do not decide on criteria for exclusion during or after the experiment.

Measures to reduce the risk of bias include randomisation, blinding, and clearly defining any exclusion criteria. Failure to report these measures is associated with overestimation of treatment effects and higher chances of false-positive results (Hirst *et al.* 2014, Macleod *et al.* 2015). Implementing these measures, however, can be resource-intensive and we recommend researchers consider any additional support required when planning and seeking funding for their experiments.

#### Randomisation

Researchers can reduce selection bias by randomly assigning animals to experimental groups using a systematic process such as a random number generator (e.g. the RAND function of Microsoft Excel) or specialised tools such as Randomice (van Eenige *et al.* 2020). Variables such as home cage position in a room or within a cage rack, the presence of litter or cage mates, and baseline measures of variables such as animal weight may also introduce bias into experiments. So-called ‘nuisance variables’ or blocking factors can be accounted for using randomisation within blocks or counterbalancing (Bate & Clark 2014).

In publications, researchers should report whether or not randomisation was performed, the type of randomisation used, and the method used to achieve randomisation (Percie du Sert *et al.* 2020). If animals were not randomly allocated to experimental groups (e.g. in studies comparing transgenic and wildtype animals), researchers should state why not and report how groups were created.

#### Blinding

Blinding is a strategy used to conceal an animal’s group allocation throughout different steps of the experiment. Allocation concealment (i.e. blinding of the group allocation) can be used alongside randomisation to minimise selection bias when allocating the animals to the experimental groups. This means that instead of researchers randomly allocating animals to, for instance, the ‘treatment’ group or ‘ontrol’ group, animals are assigned to coded group identities, for example, group A or B. Knowledge of an animal’s group allocation during husbandry and experimentation or outcome assessment can introduce performance and detection biases respectively, as a researcher’s expectations or unconscious biases may influence how they interpret animal behaviour or results. These expectations may also influence researcher behaviour, which may in turn influence animal behaviour (Rosenthal 1963). It has been previously suggested that non-blinded outcome assessors exaggerated odds ratios for the benefits or harms of an intervention over a control by an average of 59% relative to blinded outcome assessors across a variety of animal experiments measuring potentially subjective biological outcomes (Bello *et al.* 2014).

In publications, researchers should report whether or not blinding was used, at which stages of the experiment researchers were blinded, and which condition(s) or intervention(s) they were blinded to (Percie du Sert *et al.* 2020). Where researchers were not blinded (e.g. in transgenic studies with different phenotypes), reports should state the reason.

#### Reporting exclusion criteria

During animal experiments, researchers may, appropriately, need to exclude a particular animal, experimental unit, or data point from their analysis. Exclusions can take place for many reasons, including animal characteristics (body weight or task performance not meeting a certain threshold), technical issues (surgery complications), or ethical concerns (euthanasia to prevent unnecessary harm) (Percie du Sert *et al.* 2020). However, systematic differences between the experimental groups in how exclusions are handled can cause attrition bias skewing the results of the study. For instance, animal exclusions which are different, or are applied differently, between the treatment and control group, can bias experimental results.

Exclusion criteria should be defined *a priori* and reported during the planning stages (Percie du Sert *et al.* 2020). If possible, decisions on which animals or data points to exclude should be made blind to group allocation (Percie du Sert *et al.* 2020). Where an exclusion is necessary for reasons that were not envisaged during study design, the reason for exclusion should be reported.

Similarly, there may be an interaction between the intervention and exclusions. Using the example of preclinical stroke research, if an intervention leads to death in animals with large volumes of cerebral infarcts, surviving animals in that group will have smaller infarcts, and the analysis may falsely show a beneficial effect of the intervention on infarct size compared with the control group, in which animals with larger infarcts survived. It is therefore essential to transparently report all exclusions of animals, samples, or data points separately for each experimental group, including all reasons for exclusion.

It may be justifiable to exclude outliers from an analysis if this is due to factors which are clearly unrelated to the natural variation in the population, such as technical failure or a recording error, but abnormally small or large values should not be excluded simply because they did not fall within the expected range of values. The criteria for the definition of an outlier and when they are to be excluded from the dataset should be defined prior to the experiment and not decided upon during data inspection, and the exclusion of any data points should be adequately justified and reported and should be performed blinded to group identity (i.e. be aware of only coded alphanumeric identifiers, not Disease/Treatment or Control groups).

### Appropriate sample sizes and statistical power

Animal studies regrettably tend to use fewer animals than required for robust conclusions to be drawn. In the context of substantial publication bias, this can lead to a preponderance of false-positive, irreproducible claims in the research literature. In an experiment, the sample size (denoted as N) represents the total number of experimental units present in each experimental group (individually n, such that Σn = N) (Lazic *et al.* 2018). The experimental unit is defined as the smallest entity within an experiment which can be subjected to a treatment or intervention independently of all other experimental units, in other words, the smallest unit at which a ‘subject’ can be randomised to an intervention. For instance, the experimental unit may be the individual animal (e.g. in experiments where a drug is administered to individual animals), a litter (e.g. where an intervention is administered to a dam and investigated in her pups), or a cage (e.g. where animals within a cage are all fed a specific experimental diet, such as a high-fat diet, as an intervention).

Researchers need to be able to correctly identify the experimental unit – and therefore the *n* number of each of their experimental groups – both prior to conducting their experiment (to ensure their sample size is large enough), and prior to data analysis (to ensure data are analysed appropriately). Pseudoreplication, where the reported sample size is artificially inflated, is a common issue that may produce false-positive results and violates the assumptions of statistical tests used (Lazic 2010, Eisner 2021, Bannach-Brown *et al.* 2022).

A sample size calculation should be performed before the experiment is performed to determine the sample size likely to detect an effect of a given size if indeed there is a true effect to detect (Herzog *et al.* 2019). Without a sample size calculation, in the case of negative results, it becomes difficult to distinguish where a study was not sufficiently powerful to detect an effect (false negative), or where an effect was truly absent, often leading to incorrect conclusions regarding the hypothesis. A 2015 study in a random sample of life sciences articles indexed in PubMed found that none out of the 146 studies assessed reported a sample size calculation (Macleod *et al.* 2015).

We do not recommend basing the sample size on those used in previous studies, as these studies may have been underpowered. Sample size calculations should be performed *a priori* using effect size estimates from previous experiments, a pilot study, or historical data from published literature to estimate effect sizes (Bate 2018) – although effect sizes in replication studies are usually substantially smaller than in the original study (Errington *et al.* 2021). An alternative approach is to consider the minimum effect size of biological importance and power of a study to detect this effect. There are many tools available to help researchers perform a sample size calculation, including the Experimental Design Assistant tool developed by the NC3Rs (Percie du Sert *et al.* 2017). However, we recommend asking an applied statistician for help if in doubt. The NC3Rs have also published a blog on how to decide the appropriate sample size when a sample size calculation is not straightforward (Bate 2018).

In publications, researchers should report how the sample size was determined. If no calculation was used, researchers must explicitly state how the sample size was decided and provide reasoning to explain why this approach was taken.

### Further considerations for experimental design: complexity, planning, biological variables, and controls

A wide range of variables may determine real differences in outcomes measured between experimental groups. More complex experimental designs may help understand the role of multiple experimental variables but create additional complexity in experimental procedures and data analysis, making the experiment more vulnerable to procedural, data collection, and data analysis errors. Complex experimental design must also be more rigorous, requiring meticulous planning and record keeping. A complex multi-factorial design may sometimes be more appropriate and be of sufficient statistical power but increases the demands of experimental design and often the number of experimental animals required simultaneously and associated experimental costs. A simpler and more optimised design may also be associated with greater statistical power (Herzog *et al.* 2019), and the findings will generally be easier to interpret.

Prior to starting a larger study, particularly in the case of studies with more complex designs or those with many unknown variables, carrying out a smaller pilot, also known as a feasibility study, can be useful to ensure that the chosen experimental design can be implemented and that the experimental procedures function as intended. A pilot study allows the collection of experimental data prior to the investment of significant numbers of animals, funding, and other resources and ultimately can inform the design and improve the quality of the main experiment (https://nc3rs.org.uk/3rs-resources/conducting-pilot-study).

Several biological and technical variables may have a strong influence on study outcomes yet are underreported or often not considered when designing an experiment; these include animal sex, weight, age, genetic strain, housing characteristics, or comorbidities. These variables may influence the severity of a disease model, the response to a treatment, or basic biological functions within an animal model and as such may account for an amount of heterogeneity in a response observed within a study or differences observed between studies (https://grants.nih.gov/policy/reproducibility/guidance.htm). In particular, animal sex has gained attention as influential across a variety of biological traits (International Mouse Phenotyping Consortium *et al.* 2017). Consequently, the National Institutes of Health expects that animal sex be considered a key variable of interest, to be included as a factor in experimental design and statistical analyses, and be reported appropriately. Additionally, researchers should be aware of the importance of health monitoring and pathogen detection in laboratory animals and the impact that pathogens can have on animal physiology and behaviour (Buchheister & Bleich, 2021).

The ARRIVE guidelines (Percie du Sert *et al.* 2020) also highlight the importance of using a correct control group for an experiment. This design choice depends on the objectives of the proposed research, with positive and negative controls serving different purposes. The use of an appropriate negative control (e.g. a sham surgical procedure or injection of a vehicle for a pharmacological intervention) ensures that any difference observed between treatment and control groups is likely due to the treatment itself, rather than the administration procedure.

Finally, researchers should report any standardisation of procedures used, for instance, having all surgeries performed by the same surgeon to increase uniformity and reduce variability.

### Tools to help design and report experiments

Several tools and guidelines are available to support preclinical researchers in planning and reporting animal experiments. A common thread through each of these resources is their focus on transparency and detailed reporting as key requirements for planning and producing reproducible research. A selection of available resources is described in more detail later and summarised in Table 2.

**Table 2:** Summary table of tools available to support researchers in planning and reporting reproducible animal research.

Tool or guideline	Year	Purpose	Description
EQIPD quality system (Bespalov *et al.* 2021)	2021	All phases	A set of 18 essential recommendations aiming to improve the reproducibility and reliability of preclinical research.https://quality-preclinical-data.eu/about-eqipd/eqipd-quality-system/
The Experimental Design Assistant (Percie du Sert *et al.* 2017)	2017	Planning	The EDA is an online platform facilitating researchers to plan animal experiments, get feedback on their study design, and download a schematic diagram, which can be used to visualise their study design and improve reproducibility and transparency. This resource aims to help improve internal validity and animal welfare alongside reducing bias and research waste. https://eda.nc3rs.org.uk/
The PREPARE guidelines (Smith *et al.* 2018)	2018	Planning	15-item checklist aiming to improve the planning of animal research, embedding reproducibility from the very beginning. PREPARE covers three broad areas which they claim are often missing from other reporting guidelines: (i) formulation of the study, (ii) dialogues between scientists and the animal facility, and (iii) methods, including health monitoring, housing and husbandry, and humane killing, release, reuse, or rehoming. https://norecopa.no/prepare
The ARRIVE guidelines (Percie du Sert *et al.* 2020)	2010 (updated in 2020)	Reporting	A checklist of 10 ‘essential’ and 11 ‘recommended’ items, aiming to improve reproducibility, transparency, and animal welfare. Endorsed by over 1000 journals. https://arriveguidelines.org/
Gold Standard Publication Checklist (GSPC) (Hooijmans *et al.* 2010)	2010	Reporting	An extensive checklist aiming to improve transparency and animal welfare in preclinical studies. https://doi.org/10.1177/026119291003800208
The MDAR Checklist (Macleod *et al.* 2021)	2021	Reporting	18-item checklist designed to help authors comply with the MDAR (Materials, Design, Analysis, and Reporting) Framework, which may be endorsed by journals. The MDAR Framework aims to improve transparency in four key areas of life sciences research: Materials, Design, Analysis, and Reporting. https://osf.io/bj3mu/

As an additional resource, researchers may find it useful to be aware of systematic review checklists used to assess the reporting quality (Macleod *et al.* 2004) and assess the risks of bias in animal research (Hooijmans *et al.* 2014).

Currently, no universal standard for formal researcher training on reproducible experimental design and reporting exists. Some institutions may provide internal training, such as the Edinburgh University Research Optimisation Course, which was recently made compulsory for *in vivo* researchers at the institution (https://edin.ac/3zcQ0u6).

## Open research practices

Over the past decade, open research has become increasingly important to the scientific community. Open research is characterised by a set of practices which collectively aim to make scientific research more widely available and accessible, as well as more transparent and reproducible (Munafò *et al.* 2017). Table 3 provides a summary of the key points discussed in this section.

**Table 3:** Summary of the key points discussed on each open research practice topic, addressing common concerns faced by researchers.

Preregistration	Data management and sharing	Preprints
Preregistered studies are not ‘locked in’ to a particular study design. Protocol deviations are allowed as long as they are clearly explained and choices are justified.	Many journals now require data to be shared openly at the time of publication.	Many journals accept manuscripts which have been shared as a preprint – but check journal policies to be sure.
Preregistrations can be embargoed.	Keeping a well-managed data management plan will make it easier to keep track of and analyse experimental data.	The reporting quality of preprints is much the same as peer-reviewed articles (Carneiro *et al.* 2020).

### Preregistration

A study preregistration is a permanent and public record of a proposed study design, methodology, and analysis plan, written and registered prior to the start of data collection.

Preregistration benefits science by improving transparency and helping researchers to avoid potential biases and flexible analysis procedures, including ‘hypothesising after results are known’ (HARKing) (Kerr 1998) and selective outcome reporting. HARKing can take place when researchers retrospectively determine the hypotheses to be tested based on the data obtained, instead of prior to conducting the experiment. HARKing often goes hand in hand with reframing exploratory data analyses as being hypothesis-testing. Generating hypotheses based on data obtained and then using hypothesis-testing statistical analyses violates statistical assumptions and can lead to false positive results. Selective outcome reporting occurs when the likelihood of outcomes being included in the publication differs depending on their statistical significance. For instance, when only outcome measures, time points, or experimental groups with favourable results are reported, which may lead to the overestimation of treatment efficacy. A related phenomenon is outcome switching: the replacement of originally planned outcomes with alternatives which are more appealing in terms of statistical significance. Both selective outcome reporting and outcome switching are more difficult to detect in studies that have not undergone preregistration.

Contrary to some researchers’ beliefs, preregistration does not ‘lock’ researchers into using a specific method or analysis or remove any flexibility and adaptability to unanticipated challenges. After preregistering a study, the final methods and analyses can be changed or modified from the original preregistration, as long as those changes are adequately documented and later justified. Further, it does not prohibit performing exploratory analyses with collected data but rather encourages researchers to declare when they are doing so.

By preregistering a study, researchers are prompted to more carefully consider their experimental design prior to data collection and gain an increased awareness of the measures they can take to reduce the risk of bias including randomisation and blinding, as well as improving the statistical analysis of their data (van der Naald *et al.* 2022). Researchers can choose to submit preregistrations to a variety of platforms, including general platforms such as the Open Science Framework (OSF: https://osf.io/) or dedicated preclinical platforms such as PreclinicalTrials (https://preclinicaltrials.eu (van der Naald *et al.* 2022)) and The Animal Study Registry (https://www.animalstudyregistry.org/ (Bert *et al.* 2019)) launched in 2018 and 2019, respectively. Using dedicated preclinical preregistration platforms allows work to be easily findable and their tailored protocol templates encourage researchers to evaluate their study designs prior to beginning their experiments, therefore reducing research waste, improving animal welfare, and increasing internal validity.

If ‘scooping’ of a study design or hypothesis (i.e. another researcher copying the research idea and publishing before the preregistering scientist) is a concern, some platforms allow preregistrations to be embargoed until a specified date. In the case of duplicate studies, the existence of such preregistration can then be used to assert that the preregistering investigator conceived the idea independently.

### Data management and sharing

Data sharing has become an important consideration for researchers. Many funder policies and journal guidelines now encourage or mandate data that be made publicly available where possible. Sharing data allows for other researchers to independently verify the original analysis, as well as using the data to perform further or alternative analyses using the same dataset, generating new research without the requirement to repeat the data collection process. When sharing research data, it is important to follow the FAIR principles by making datasets ‘Findable, Accessible, Interoperable, and Reusable’ (Wilkinson *et al.* 2016). The authors (Wilkinson *et al.* 2016) provide guidance on meeting these four principles.

While planning research, it is useful to consider what types of data the study will generate, how the data will be stored and managed, and how the data will be shared. These considerations can be best addressed by writing a data management plan (https://doi.org/10.1038/d41586-018-03065-z).

Some funders now require the submission of a data management plan alongside funding proposals. Even if a data management plan is not a funder or institutional requirement, the benefits of having one outweigh the work required.

Keeping a detailed, well-managed record of how research data will be collected and stored aids to prevent the loss of important data. Additionally, writing meta-data to describe datasets can help researchers remember exactly what their data mean and how and where they was collected. Meta-data is especially useful when handing over projects to other researchers or when preparing for publication.

At the point of publication and data sharing, a well-managed and documented dataset is much more understandable and easier for other researchers to reuse. Finally, data shared in public repositories can often be assigned a DOI (digital object identifier) and can be cited.

### Preprints

A preprint is a non-peer-reviewed version of a scientific publication, uploaded to an online repository by the authors and made publicly available (Kirkham *et al.* 2020). Posting research outputs as preprints allows rapid access to information, peer review, feedback from a wider community of individuals, and increased visibility.

Preprints can also help avoid publication bias, a phenomenon where positive, ‘novel’ results are more likely to be published than null or negative findings by allowing researchers to share their work regardless of their results. The implications of publication bias are the potential to greatly overstate the significance of the effect of an experimental condition or treatment in a given research field or subfield (Sena *et al.* 2010). Limiting the impact of publication bias by means of preprinting has the potential to improve transparency and reproducibility and reduce research waste, both that associated with the given study and later resulting studies.

Preprints are typically posted at the time of submission to a journal. Although most journals will publish work which has been preprinted, we advise that researchers check journal preprint policies, often found on publisher websites, to make sure that preprinting will not affect the likelihood of acceptance of their work in a scientific journal. Additionally, Sherpa Romeo is a service which researchers can use to check open access and preprint policies of scientific journals (https://v2.sherpa.ac.uk/romeo/about.html). Posting manuscripts to a preprint repository has many benefits, including making results available more promptly.

Many preprint repositories are available to host biomedical studies including Open Science Framework Preprints (https://osf.io/preprints/), bioRxiv (https://www.biorxiv.org/), and Research Square (https://www.researchsquare.com/).

Sceptics of the preprint movement worry that the rise of preprints may dilute the scientific record with unreliable science. However, a 2020 study of biomedical literature found an insignificant difference between the reporting quality of preprints vs peer-reviewed articles, with preprints performing only marginally worse (Carneiro *et al.* 2020). Additionally, preprinting manuscripts is thought to improve the final peer review of the work by enabling comments and feedback from other researchers who read the preprint (Desjardins-Proulx *et al.* 2013).

## Conclusions: considerations for now and for the future

Substantial progress has been made towards developing support for researchers conducting animal experiments to ensure that research is robust and reproducible. Much of the work we have described has been led by government agencies dedicated to improving animal welfare, such as the NC3Rs, who developed the ARRIVE guidelines and the Experimental Design Assistant (EDA); the German Centre for the Protection of Laboratory Animals (Bf3R), who developed the Animal Study Registry; the Dutch Ministry for Health, Welfare and Sport, who funded the development of the GSPC and SYRCLE's risk of bias tool; and Norecopa and the RSPCA, who developed the PREPARE guidelines.

A critical component in the creation of these tools and guidelines has been effective collaboration with members of the animal research community. Researchers are crucial stakeholders and should feel empowered to be involved in the development and testing of such resources to ensure that they are fit for purpose. Fundamentally, researchers should feel that resources aiming to improve the reproducibility of animal research are designed to support them, not hinder, restrict, or cause unnecessary burdens. In future, we seek further and stronger engagement with preclinical researchers to help improve awareness and uptake of these resources.

For now, it is important to note when considering the adoption of reproducible research practices and associated improvements to research integrity that many of these issues stem from systemic flaws in our research culture, rather than flaws of individual researchers (Macleod & The University of Edinburgh Research Strategy Group 2022). While individual researchers, especially those early in their career, may feel unable to make impactful changes to existing harmful research culture, every step towards greater reproducibility is an important step in the right direction and should be rewarded and encouraged. We also encourage research institutions to take charge in providing formal training on working reproducibly and incentivising reproducibility over novelty.

## Declaration of interest

The authors declare no conflicts of interest.

## Funding

EW is funded by a Simons Initiative for the Developing Brainhttp://dx.doi.org/10.13039/501100015504 (SIDB) PhD studentship (SFARI #529085). FR was funded by the Biotechnology and Biological Sciences Research Councilhttp://dx.doi.org/10.13039/501100000268 (BBSRC) (grant number BB/M010996/1).

## Author contribution statement

EW and FJR contributed equally to conceptualising the review, preparing the first draft, and editing subsequent versions. KEW, MRM, ESS, and GLC provided expert insights and contributed to revisions of this manuscript. MRM, ESS, and GLC provided supervision.
